# The Life Orientation Test: A Confirmatory Factor Analysis Across Three Age Groups

**DOI:** 10.1093/geroni/igaa057.1486

**Published:** 2020-12-16

**Authors:** Robert Intrieri, Paige Goodwin

**Affiliations:** Western Illinois University, MACOMB, Illinois, United States

## Abstract

The Life Orientation Test (LOT; Scheier & Carver, 1985) was developed as a measure of dispositional optimism. Optimism has been linked to positive life outcomes and is associated with psychological (Carver & Gaines, 1987; Scheier & Carver, 1985) and physical (Scheier & Carver, 1987; Scheier et al. 1989) well-being. The current study assessed 520 people placed into three age groups: young adult (n =149), middle-age adult (n = 252), and older adult (n = 119). The mean age for the young group was 19.24 (SD = 2.01), middle-aged (47.68 (SD = 4.75), and old was 71.99 (SD = 7.21). Data were submitted for a confirmatory factor analysis (CFA) which tested for invariance across the age groups. Previous factor analyses have identified two distinct factors representing optimism and pessimism. Results from the CFA showed that both Configural and Metric invariance models demonstrated acceptable fit for the two factor model (□2(df=57) = 61.92, p = 0.3047; □2(df=69) = 78.77, p = 0.1974). In contrast, Scalar invariance resulted in a poor fit across the three age groups (□2(df=81) = 139.288, p < 0.0001). Model comparisons revealed no significant differences between Configural and Metric models (□2(df=12) = 16.996, p = 0.1498). Model comparisons between Configural and Scalar and Metric and Scalar were (□2(df=24) = 78.947, p < 0.0001; □2(df=12) = 61.764, p < 0.0001). These results confirm previous research that shows a correlated two factor model consistent with the concept that optimism and pessimism are correlated elements rather than two ends of a continuum.

